# Time to Achieve a Minimal Clinically Important Difference After Total Hip Arthroplasty: A Retrospective Cohort Comparison of Robotic-Assisted, Navigation-Assisted, and Conventional Techniques

**DOI:** 10.1016/j.artd.2025.101902

**Published:** 2025-11-08

**Authors:** Kareem Omran, Colleen Wixted, Daniel Waren, Joshua C. Rozell, Ran Schwarzkopf

**Affiliations:** a Department of Public Health and Primary Care, University of Cambridge, Cambridge, UK; b Department of Orthopedic Surgery, NYU Langone Health, New York, NY, USA

**Keywords:** Total hip arthroplasty (THA), Robotic-assisted THA (RA-THA), Navigation-assisted THA (NA-THA), Minimal Clinically Important Difference (MCID), HOOS-JR, Time-to-event analysis

## Abstract

**Background:**

Technological advancements in total hip arthroplasty (THA), including robotic-assisted (RA-THA) and navigation-assisted (NA-THA) techniques, aim to improve outcomes. However, impact on recovery timing remains unclear. This study examined whether these technologies reduce the time to reach the minimal clinically important difference (MCID) on the Hip Disability and Osteoarthritis Outcome Score for Joint Replacement compared with conventional THA.

**Methods:**

This retrospective study analyzed osteoarthritic THA patients (01/2020-04/2023) who completed preoperative and postoperative Hip Disability and Osteoarthritis Outcome Score for Joint Replacement questionnaires. The exclusion criteria included bilateral procedures or revision within 1 year. MCID was defined using anchor-based (23 points) and distribution-based thresholds (7.6 points). Multivariable interval-censored accelerated failure time models assessed time to MCID.

**Results:**

Among the 1395 patients, 181 (12.9%) underwent RA-THA, 754 (54.1%) underwent NA-THA, and 460 (33.0%) underwent conventional THA. Anchor-based MCID rates were 65.2%, 63.4%, and 66.5%, respectively (*P* > .05), with median times of 38.9, 48.4, and 45.1 days. Neither RA-THA (time ratio [TR] = 0.86, 95% confidence interval [CI]: 0.63-1.18, *P* = .347) nor NA-THA (TR = 1.07, 95% CI: 0.87-1.32, *P* = .502) significantly affected time to MCID vs conventional distribution-based thresholds yielded higher MCID rates (93.9%, 88.9%, 89.8%; *P* > .05) with median times of 8.6, 11.4, and 12.9 days, respectively. RA-THA reached MCID 33.5% faster than conventional THA (TR = 0.66, 95% 26 CI: 0.52-0.86, *P* = .002) and 24.3% faster than NA-THA (TR = 0.76, 95% CI: 0.60-0.95, *P* = .019), while NA-THA showed no significant difference vs conventional THA (TR = 0.88, 95% CI: 0.74-1.04, *P* = .140).

**Conclusions:**

Anchor-based MCID demonstrated comparable recovery times across RA, NA, and conventional THA, suggesting no patient-perceived advantage with technology. Distribution-based thresholds indicated RA-THA achieved faster statistically significant improvement, though the relevance remains uncertain.

## Introduction

Total hip arthroplasty (THA) has undergone significant advancements since its inception, with innovations aimed at improving surgical precision and patient outcomes. Traditional THA techniques rely on surgeon expertise, leading to variability in implant positioning and alignment [1]. Component malposition can result in impingement, leg-length discrepancy, dislocation, and implant failure, potentially requiring revision and diminishing quality of life [2,3]. To address these challenges, robotic-assisted (RA-THA) and navigation-assisted (NA-THA) technologies have been developed. The most common RA-THA platform leaves the surgeon in ultimate control while incorporating preoperative imaging of the patient’s pelvis and hip to improve the accuracy of the femoral neck cut, acetabular reaming, and subsequent implant placement. These systems provide real-time feedback, allowing for careful adjustments in variables such as acetabular cup inclination, and anteversion, as well as leg length, and femoral offset that have been shown to contribute to hip stability and longevity [4]. Similarly, NA-THA employs computer-guidance to assist surgeons in achieving precise implant alignment [5]. Both techniques aim to improve the biomechanical outcomes of the surgery, potentially leading to better long-term results.

In assessing the success of these surgical innovations, the Minimal Clinically Important Difference (MCID) may serve as a useful tool. The MCID reflects the smallest improvement that patients perceive as meaningful, making it a valuable measure in total joint replacement surgery, where the goals are pain relief, functional restoration, and enhanced quality of life. The Hip Disability and Osteoarthritis Outcome Score for Joint Replacement (HOOS-JR), a validated patient-reported outcome measure (PROM), is instrumental in evaluating these outcomes, enabling the quantification of an MCID. Beyond simply attaining an MCID, the speed at which this threshold is reached is equally important, as faster recovery may enhance patient satisfaction, reduce health-care burdens, and allow for quicker return to daily activities.

While numerous previous research has explored MCID attainment rates across different surgical approaches [[6], [7], [8], [9]], the time to reach this threshold remains underinvestigated. As such, this study aimed to answer the following questions: (1) are the proportions of patients achieving an MCID different in the first 12 months postoperatively between RA-THA, NA-THA, and conventional THA? and (2) is there a difference in the timing of MCID attainment between RA-THA, NA-THA, and conventional THA?

## Material and methods

### Study design and data collection

This retrospective cohort study included all patients undergoing primary THA for osteoarthritis between January 1, 2020, and April 1, 2023, at a tertiary academic orthopaedic hospital. Exclusion criteria included patients undergoing THA for indications other than osteoarthritis, those who did not have a baseline HOOS-JR or at least 1 postoperative HOOS-JR within the first year, bilateral procedures, and those who underwent revision surgery within 1 year. Patient demographics and clinical characteristics collected included sex, race, age, marital status, body mass index, smoking status, American Society of Anesthesiologists score, procedure type (conventional, NA-THA, or RA-THA), type of anesthesia, and discharge disposition. The HOOS-JR questionnaires were administered preoperatively and at postoperative office visits as part of routine clinical follow-up at 1, 3, 6, and 12 months. Only questionnaires within the first 12 months postoperatively were included. As patients often had multiple visits preoperatively, baseline scores were calculated as the mean of all preoperative scores within the 6 months prior to surgery.

### Outcome measure and sensitivity analysis

The primary outcome was the time to achieve an MCID in HOOS-JR scores, which range from 0 to 100, with higher scores indicating better hip function and lower scores indicating greater hip disability. Given the absence of a universally applicable MCID threshold, we utilized 2 methods to define it: an anchor-based threshold of 23 points, derived from a comparable patient cohort, and a distribution-based threshold of 7.6 points, calculated as half the standard deviation of baseline scores in our cohort [[10], [11], [12]].

### Patient population

**Figure 1:**
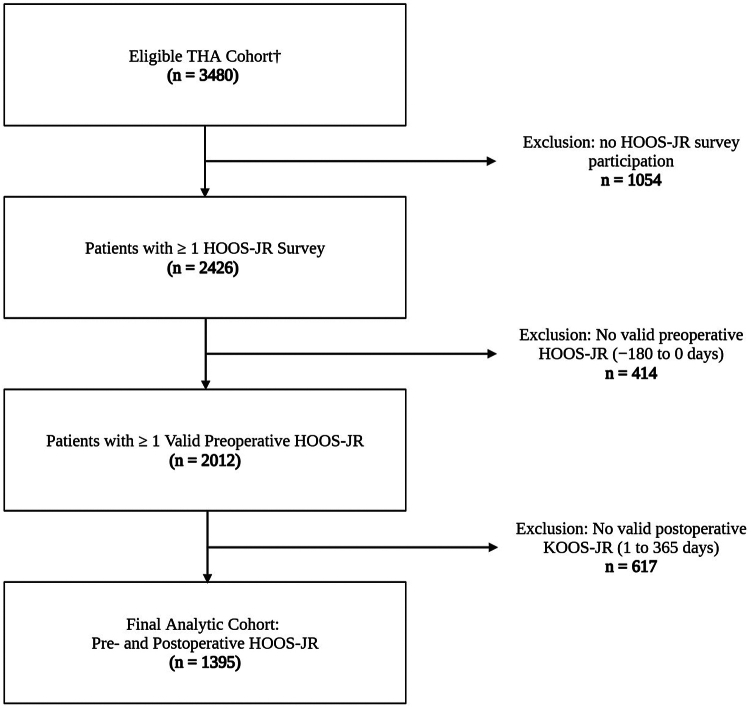
Flowchart illustrating the inclusion and exclusion criteria for the analytic cohort of patients undergoing primary THA with complete preoperative and postoperative HOOS-JR data (n = 1395).

Out of 3480 patients who underwent elective primary unilateral THA procedures in the study period, 1395 patients met the inclusion criteria based on availability of PROM scores (Fig. 1). The cohort had a median age of 67 years (interquartile range (IQR): 60-73), a median body mass index of 28.9 (IQR: 25.3-33.4) with 58.1% being women. Data completeness was high, with no missing values for any covariate. The cohort included 181 patients (12.9%) who underwent RA-THA, 754 (54.1%) NA-THA, and 460 (33.0%) conventional THA (Table 1). All RA-THA cases were performed with the Mako robotic system (Stryker, Kalamazoo, MI). In the NA-THA cohort, 486 of 754 procedures (64.4 %) used the Intellijoint navigation system (Intellijoint Surgical Inc., Kitchener, ON, Canada) and 268 (35.6 %) used the Radlink navigation system (Radlink, El Segundo, CA). Sensitivity analyses stratified by navigation platform demonstrated no significant differences in outcomes, supporting their pooling for analysis.

**Table 1:** Patient demographics and clinical characteristics by procedure type (N = 1395).

Characteristic	Conventional THA, N = 460^a^	Navigation-assisted THA, N = 754^a^	Robotic-assisted THA, N = 181^a^
Preoperative HOOS-JR Score	50 (40, 59)	50 (40, 58)	48 (38, 57)
Age at surgery	68 (63, 74)	66 (59, 72)	65 (58, 72)
Sex			
Women	263 (57%)	441 (58%)	106 (59%)
Men	197 (43%)	313 (42%)	75 (41%)
BMI	28.4 (24.9, 32.9)	29.0 (25.3, 33.0)	30.2 (26.5, 35.7)
Race			
White	370 (80%)	562 (75%)	118 (65%)
Black or African American	58 (13%)	84 (11%)	30 (17%)
Asian	8 (1.7%)	19 (2.5%)	6 (3.3%)
Other	21 (4.6%)	85 (11%)	25 (14%)
Undeclared	3 (0.7%)	4 (0.5%)	2 (1.1%)
Marital Status			
Currently partnered	263 (57%)	437 (58%)	103 (57%)
Currently single	146 (32%)	261 (35%)	69 (38%)
Widowed	51 (11%)	56 (7.4%)	9 (5.0%)
Smoking Status			
Never	232 (50%)	419 (56%)	89 (49%)
Current	16 (3.5%)	46 (6.1%)	8 (4.4%)
Former	212 (46%)	289 (38%)	84 (46%)
ASA physical status			
1-2	298 (65%)	551 (73%)	119 (66%)
3-4	162 (35%)	203 (27%)	62 (34%)
Anesthesia type			
Regional	429 (93%)	688 (91%)	168 (93%)
General	31 (6.7%)	66 (8.8%)	13 (7.2%)
Discharge disposition			
Home	437 (95%)	728 (97%)	178 (98%)
Facility-based care	23 (5.0%)	26 (3.4%)	3 (1.7%)

BMI, body mass index; ASA, American Society of Anesthesiologists.

a Median (IQR); n (%).

The overall mean preoperative HOOS-JR score was 47.9 (standard deviation [SD] 15.2). Mean scores by procedure type were 46.7 (SD 16.0) for RA-THA, 48.1 (SD 15.1) for NA-THA, and 48.0 (SD 15.0) for conventional THA. Ceiling effects were minimal; only 2.4% (3 of 1395) of patients had preoperative HOOS-JR scores high enough to mathematically preclude reaching the anchor-based MCID threshold, and none of the patients for the distribution-based threshold.

Patients completed a median of 2 postoperative questionnaires (IQR 1-3). The first questionnaire occurred a median of 20 days after surgery (IQR 15-44), and the last at 104 days (IQR 57-204).

### Data analyses

Accelerated failure time (AFT) models evaluated time to MCID. The log-logistic distribution was utilized in the AFT models based on Akaike Information Criterion values and best clinical fit (Supplementary Table 1). Since an MCID can be achieved between questionnaire completions, interval censoring was applied to provide a more accurate estimate of MCID achievement. The interval to MCID was defined as the time between the questionnaire that first met the MCID threshold and the preceding questionnaire, or from postoperative day 1 if no earlier measure was available. For patients who did not achieve MCID during the follow-up period, right-censoring was applied at the last available questionnaire.

Time ratios (TRs) quantified associations between procedure type, patient characteristics, and time to MCID. Covariate-standardized median times to MCID were estimated using parametric G-computation, with 95% confidence intervals (CIs) derived from 1000 parametric bootstrap resamples. Sensitivity analyses additionally adjusted for surgeon-preferred surgical approach (posterior, anterior, direct lateral, or mixed), with results provided in the Supplementary Material. Approach was defined by each surgeon’s predominant practice pattern, as case-level operative notes were not available. Surgeons who regularly performed both anterior and posterior approaches were categorized as “mixed,” which reduced the risk of bias that could arise if selective approach choice were related to patient comorbidity or case complexity. Inclusion of approach did not materially alter the main associations or improve model fit, and therefore it was not retained in the primary models.

### Missing data and bias

To assess potential biases, 2 additional descriptive analyses were performed. Selection bias was evaluated by comparing baseline characteristics between the included cohort and patients who underwent primary unilateral THA for osteoarthritis at the same institution during the study period but were excluded from the analysis (Table 2). Attrition bias was assessed by comparing patients who completed at least 1 preoperative and 1 postoperative HOOS-JR assessment ("Responders") to those who did not provide postoperative data ("Non-responders") (Table 3).

**Table 2:** Comparison of baseline patient characteristics between the final analyzed cohort and the excluded THA cohort (N = 3480).

Characteristic	Analyzed cohort, N = 1,395^a^	Excluded THA cohort, N = 2,085^a^
Procedure type		
Conventional THA	460 (33%)	730 (35%)
Navigation-assisted THA	754 (54%)	852 (41%)
Robotic-assisted THA	181 (13%)	503 (24%)
Age at surgery	67 (60, 73)	66 (59, 74)
BMI	28.9 (25.3, 33.4)	28.6 (25.1, 32.9)
Sex		
Women	810 (58%)	1141 (55%)
Men	585 (42%)	944 (45%)
Marital status		
Currently partnered	803 (58%)	1167 (56%)
Currently single	476 (34%)	706 (34%)
Widowed	116 (8.3%)	212 (10%)
Smoking status		
Never	740 (53%)	1142 (55%)
Current	70 (5.0%)	157 (7.5%)
Former	585 (42%)	786 (38%)
ASA physical status		
1-2	968 (69%)	1358 (65%)
3-4	427 (31%)	727 (35%)
Race		
White	1050 (75%)	1584 (76%)
Black or African American	172 (12%)	266 (13%)
Asian	33 (2.4%)	33 (1.6%)
Other	131 (9.4%)	194 (9.3%)
Undeclared	9 (0.6%)	8 (0.4%)
Anesthesia type		
Regional	1285 (92%)	1871 (90%)
General	110 (7.9%)	214 (10%)
Discharge disposition		
Home	1343 (96%)	1964 (94%)
Facility-based care	52 (3.7%)	121 (5.8%)

BMI, body mass index; ASA, American Society of Anesthesiologists.

a n (%); Median (IQR).

**Table 3:** Comparison of baseline patient characteristics between responders and nonresponder cohorts (N = 2012).

Characteristic	Responders, N = 1,395^a^	Nonresponders, N = 617^a^
Procedure type		
Conventional THA	460 (33%)	212 (34%)
Navigation-assisted THA	754 (54%)	217 (35%)
Robotic-assisted THA	181 (13%)	188 (30%)
Age at surgery	67 (60, 73)	65 (59, 72)
BMI	28.9 (25.3, 33.4)	28.2 (25.0, 32.6)
Sex		
Women	810 (58%)	342 (55%)
Men	585 (42%)	275 (45%)
Marital status		
Currently partnered	803 (58%)	366 (59%)
Currently single	476 (34%)	205 (33%)
Widowed	116 (8.3%)	46 (7.5%)
Smoking status		
Never	740 (53%)	346 (56%)
Current	70 (5.0%)	44 (7.1%)
Former	585 (42%)	227 (37%)
ASA physical status		
1-2	968 (69%)	423 (69%)
3-4	427 (31%)	194 (31%)
Race		
White	1050 (75%)	491 (80%)
Black or African American	172 (12%)	64 (10%)
Asian	33 (2.4%)	15 (2.4%)
Other	131 (9.4%)	44 (7.1%)
Undeclared	9 (0.6%)	3 (0.5%)
Anesthesia type		
Regional	1285 (92%)	556 (90%)
General	110 (7.9%)	61 (9.9%)
Discharge disposition		
Home	1343 (96%)	593 (96%)
Facility-based care	52 (3.7%)	24 (3.9%)

BMI, body mass index; ASA, American Society of Anesthesiologists.

a n (%); Median (IQR).

### Ethical approval and data analysis tools

Patient records were deidentified under our institutional quality improvement program, with the study reviewed and deemed exempt by the institutional review board (study number: i20-01700). Data analyses was performed using RStudio (version 4.2.2), with statistical significance set at the 5% level. Reporting adhered to the Strengthening the Reporting of Observational Studies in Epidemiology guidelines [13].

## Results

### MCID attainment rates

Using the anchor-based method, 65.2% of RA-THA, 63.4% of NA-THA, and 66.5% of conventional THA patients achieved an MCID within the first year. A *Pearson’s chi-squared* test found no significant difference between groups (*X*^2^ = 1.25, df = 2, *P* = .536). The distribution-based method showed higher MCID attainment rates: 93.9% for RA-THA, 88.9% for NA-THA, and 89.8% for conventional, with no significant difference (*X*^2^ = 4.09, df = 2, *P* = .129).

### Time to MCID by procedure type

Table 4 summarizes the associations between patient and procedural characteristics with time to MCID using both anchor-based and distribution-based thresholds.

**Table 4:** TRs for Achieving a MCID in HOOS-JR Scores by patient and procedure characteristics using distribution- and anchor-based MCID thresholds.

Variable	Distribution-based threshold			Anchor-based threshold		
	TR	95 % CI	*P* value	TR	95 % CI	*P* value
Preoperative HOOS-JR Score	1.04	1.03-1.04	**<.001**	1.07	1.06-1.08	**<.001**
Procedure type						
Conventional THA (reference)						
Navigation-assisted THA	0.88	0.74-1.04	.140	1.07	0.87-1.32	.502
Robotic-assisted THA	0.66	0.52-0.86	**.002**	0.86	0.63-1.18	.347
Age at surgery	1.01	1.00-1.02	**.011**	1.01	1.00-1.02	**.015**
BMI	1.01	1.00-1.02	.153	1.00	0.99-1.02	.845
Sex						
Women (reference)						
Men	0.95	0.81-1.11	.503	0.85	0.70-1.04	.112
Marital status						
Currently partnered (reference)						
Currently single	1.08	0.91-1.28	.361	1.06	0.87-1.31	.556
Widowed	0.95	0.71-1.28	.755	0.82	0.58-1.17	.283
Smoking status						
Never smoked (reference)						
Current smoker	1.27	0.90-1.79	.182	1.27	0.83-1.95	.277
Former smoker	1.04	0.89-1.22	.618	1.11	0.91-1.34	.305
ASA physical status						
1-2 (reference)						
3-4	0.90	0.75-1.07	.225	1.03	0.83-1.28	.786
Race						
White (reference)						
Black or African American	1.24	0.97-1.58	.092	1.34	0.99-1.80	.057
Asian	1.69	1.05-2.74	**.032**	1.26	0.67-2.37	.470
Other	1.06	0.81-1.40	.661	1.15	0.82-1.60	.415
Undeclared	0.86	0.34-2.19	.753	0.84	0.28-2.51	.753
Anesthesia type						
Regional (reference)						
General	1.25	0.94-1.66	.121	1.28	0.91-1.81	.161
Discharge disposition						
Home (reference)						
Facility-based care	1.76	1.15-2.67	**.009**	1.15	0.68-1.93	.608

BMI, body mass index; ASA, American Society of Anesthesiologists.

Bolded *P* values indicate statistical significance (*P* < .05).

#### Anchor threshold

Neither RA-THA (TR = 0.86, 95 % CI: 0.63-1.18, *P* = .347) nor NA-THA (TR = 1.07, 95 % CI: 0.87-1.32, *P* = .502) differed in time to achieve an MCID from conventional THA. After covariate standardization, the model-estimated median time to MCID was 38.9 days (95 % CI: 29.4-54.0) for RA-THA, 48.4 days (95 % CI: 41.3-55.6) for NA-THA, and 45.1 days (95 % CI: 37.6-53.4) for conventional THA.

#### Distribution threshold

RA-THA reduced time to MCID by 33.5% compared to conventional THA (TR = 0.66, 95 % CI: 0.52-0.86, *P* = .002) and by 24.3% compared to NA-THA (TR = 0.76, 95 % CI: 0.60-0.95, *P* = .019). NA-THA showed no difference from conventional THA (TR = 0.88, 95 % CI: 0.74-1.04, *P* = .140). Covariate standardization yielded estimated median times of 8.6 days (95 % CI: 7.1-10.2) for RA-THA, 11.4 days (95 % CI: 10.3-12.6) for NA-THA, and 12.9 days (95 % CI: 11.1-14.9) for conventional THA.

### Impact of patient characteristics on time to MCID

#### Anchor threshold

Each 1-point increase in preoperative HOOS-JR score prolonged time to MCID by 7 % (TR = 1.07, 95 % CI 1.06-1.08, *P* < .001). Age exerted a smaller but significant effect, increasing time to MCID by 1 % per year (TR = 1.01, 95 % CI 1.00-1.02, *P* = .015). Black or African American race was associated with a 34% slower time to MCID, but this did not reach the statistical significance threshold (TR = 1.34, 95 % CI 0.99-1.80, *P* = .057). No other covariate reached statistical significance.

#### Distribution threshold

Higher preoperative HOOS-JR score (TR = 1.04, 95 % CI 1.03-1.04, *P* < .001) and older age (TR = 1.01, 95 % CI 1.00-1.02, *P* = .011) again lengthened time to MCID. Asian race was also associated with a 69% longer time (TR = 1.69, 95 % CI 1.05-2.74, *P* = .032), and discharge to facility-based care extended time by 76 % (TR = 1.76, 95 % CI 1.15-2.67, *P* = .009).

## Discussion

This study assessed the impact of technology-assisted THA on recovery times, demonstrating that the choice of MCID calculation method profoundly influences outcome interpretation. The distribution-based method, with its lower threshold, suggested that RA-THA resulted in faster MCID achievement than NA-THA and conventional THA. In contrast, the higher anchor-based threshold showed no significant advantage for RA-THA or NA-THA.

Both methods are widely used to define MCID thresholds but offer distinct insights into patient recovery. The distribution-based method provides a statistical calculation for the smallest value increase that is statistically unlikely to be due to random chance, offering an objective measure of functional improvement. However, it lacks direct clinical correlation, and whether such statistically detectable changes translate to patient-perceived benefits remains debated in the literature. Conversely, the anchor-based method ties score improvements to patient-reported experiences, making it better suited for capturing clinically relevant benefits [12]. We utilized both methods of threshold calculation for transparency and to offer a more comprehensive view of recovery after THA, as the distribution-based approach, despite its limitations, provides a statistically validated benchmark that can highlight subtle but significant treatment-related differences in PROM trajectories, thereby complementing the clinically grounded anchor-based estimates. Gold-standard recommendations prioritize deriving an anchor-based threshold within the study cohort whenever feasible; however, when an external anchor is unavailable, using a previously established threshold from a demographically similar cohort is an endorsed alternative [12]. In accordance with these guidelines, this study adopted an anchor threshold derived via receiver operating characteristic curves analysis of the patient-acceptable symptom state questionnaire, a validated PROM, from a similar high-volume orthopaedic center with a comparable patient population [10].

To our knowledge, this is the first study to analyze and compare time to MCID achievement after THA between different surgical technologies. Previous systematic reviews by Han et al., Karunaratne et al., and Lawrence et al. have demonstrated comparable outcomes for NA-THA, RA-THA, and conventional THA regarding pain relief, quality of life, and patient satisfaction [[14], [15], [16]]. However, these studies did not assess time to achieve these outcomes, which remains underexplored compared to radiological and general clinical endpoints [[6], [7], [8]]. The early statistically quantifiable improvements observed in this study with RA-THA may be attributed to the technology's enhanced accuracy and precision in implant placement. Improved acetabular cup positioning and restoration of leg length and offset may be associated with earlier mobilization and ease during therapy [15,[17], [18], [19]]. Additionally, the reduced soft tissue disruption and postoperative inflammation associated with robotic-assisted procedures may contribute to accelerated initial functional recovery [20]. Understanding the potential influencers of early recovery patterns can inform surgical decision-making, helping surgeons and patients select appropriate techniques based on individual priorities for recovery speed and long-term outcomes. However, the clinical significance of these improvements remains uncertain, as the absolute time differences were modest and most patients would likely achieve the distribution-based MCID threshold before their first follow-up assessment. Furthermore, despite these observed variations in the speed of early recovery, overall MCID attainment rates in our study were comparable across all groups, reinforcing findings from existing literature that technologies like RA-THA and NA-THA achieve similar long-term patient-reported outcomes compared to conventional approaches [[6], [7], [8], [9]].

In addition to procedural differences, several patient characteristics were observed to significantly influence the time to achieve an MCID. Patients discharged to rehabilitation facilities required more time to reach MCID than those discharged home, possibly due to differences in care intensity or continuity. Such patients might also miss out on the advantages provided by rapid recovery protocols. Further, evidence from arthroplasty literature suggests outpatient or home-based care can be equally or more effective compared to inpatient rehabilitation [[21], [22], [23]]. Higher preoperative HOOS-JR scores were also consistently associated with delayed MCID achievement, possibly reflecting the greater difficulty in achieving substantial relative improvement. While ceiling effects could theoretically account for this, the association persisted in the distribution-based analysis, where no patient’s baseline scores mathematically precluded MCID attainment, supporting a genuine clinical association [24]. Recovery times also varied by race, with Asian patients reaching MCID slower than White patients, contrasting previous literature demonstrating favorable outcomes in Asian cohorts [25]. However, the small size of our Asian cohort precludes robust conclusions regarding racial differences.

Methodological considerations also impact the interpretation of recovery patterns. Given our primary objective to evaluate how surgical techniques influence the timing of functional recovery, we utilized parametric AFT models rather than the more commonly applied Cox proportional hazards models. Unlike Cox models, which estimate instantaneous risk and do not directly reflect overall recovery speed, AFT models yield TRs that quantify how much faster or slower 1 group achieves an outcome compared to another. For example, a TR of 0.66 for RA-THA relative to conventional THA (based on distribution-based MCID) indicates that RA-THA patients reached the MCID threshold in 66% of the time required by conventional THA patients, representing a 34% faster recovery time.

AFT models also offer practical statistical advantages over Cox proportional hazard models. They are not prone to violations of the proportional hazards assumption, which are common in total joint arthroplasty research, and are better suited to handle the interval-censored data inherent in our study design [26,27]. In addition to favorable Akaike Information Criterion values, we selected the log-logistic distribution, as its distribution reflects the typical postoperative course following THA, characterized by rapid early improvement over the first 6 to 12 weeks, followed by a slowing and eventual plateau in functional gains over subsequent months [[28], [29], [30]]. This distributional alignment enhances model performance and improves the reliability of time-to-event estimates [27]. Such precision is essential for setting realistic expectations with patients, particularly given the known impact of preoperative expectations on perceived recovery and postoperative satisfaction [31,32].

Despite the study’s strengths, several limitations should be acknowledged. The use of patient-reported surveys to determine MCID introduces some uncertainty in the exact timing of functional improvement. While interval censoring methods partially mitigate this, the available PROM data were limited, with a median of 2 postoperative assessments per patient and variability in timing. This follow-up pattern may reduce the precision of time-to-MCID estimates and limit the ability to fully characterize recovery trajectories. We elected not to restrict our analysis to patients with complete questionnaire data, as such exclusion criteria could introduce selection bias by over-representing highly compliant patients, thereby limiting the generalizability of our results to the broader surgical population. Nonetheless, future prospective studies with more frequent and standardized assessments may help to refine these estimates and further clarify the role of surgical technology in accelerating functional recovery.

Additionally, surgeon distribution by procedure type was not explicitly controlled for in the analyses, and the relatively smaller cohort size for robotic-assisted procedures may have limited statistical power to detect more subtle differences in recovery patterns. However, 80% of procedures were performed by 8 high-volume surgeons, and all surgeons in this study were highly experienced and beyond the learning curve for both RA-THA and NA-THA, minimizing variability related to surgical expertise. Further, because operative notes were unavailable, surgical approach was characterized at the surgeon rather than the case level. Surgeons were classified by their predominant approach (anterior, posterior, or direct lateral), or as “mixed” if they used more than 1 technique. This definition reduced potential bias from selective approach use based on patient comorbidity but also limited sensitivity to detect approach-specific differences in recovery. Given that previous studies have reported a potentially faster early recovery with the anterior approach [[33], [34], [35], [36]], the absence of such a difference in our data should be viewed considering this methodological constraint.

Although robotic-assisted THA systems share the principle of enhanced accuracy and biomechanical restoration, all procedures in this study were performed exclusively with the Mako system (Stryker, Mahwah, NJ), which may limit generalizability to other platforms. Multicenter studies across varied populations and health-care settings would be beneficial to enhance external generalizability.

The study’s findings must also be interpreted considering potential selection and attrition biases. The analyzed cohort had more navigation-assisted procedures and fewer robotic-assisted procedures than those excluded. This pattern persisted among nonresponders, who had more robotic-assisted procedures and fewer navigation-assisted procedures than responders, suggesting potential underrepresentation of robotic-assisted outcomes. However, robotic-assisted procedures comprised 12.9% of our study cohort, which exceeds the 6.6% reported in the 2024 American Joint Replacement Registry for elective primary THA in 2023, indicating that RA-THA was not underrepresented relative to contemporary national practice patterns [37]. If patients with more severe symptoms or complex presentations were less likely to participate in PROMs or complete follow-up, the findings may disproportionately reflect patients with less complicated recovery trajectories.

## Conclusions

This study found that time to MCID was similar across procedure types when assessed using the anchor-based threshold. In contrast, the distribution-based threshold suggested that RA-THA may accelerate early functional recovery, representing a statistically significant improvement in the time to achieve a minimal increase in PROM scores that was unlikely to occur by chance, although whether this translates into a perceptible patient benefit remains uncertain. The potential value and clinical relevance of this earlier statistical based improvement warrants further investigation. These findings highlight the importance of MCID definition when interpreting clinical outcomes and emphasize the distinction between statistical significance and clinical meaningfulness. While overall MCID attainment proportions were comparable across groups, time to MCID may provide an additional dimension for evaluating procedural outcomes in THA. Future research should prioritize frequent early postoperative data collection to determine whether technology-assisted THA offers meaningful advantages for patients seeking faster recovery.

## Conflicts of interest

Joshua C. Rozell is on the speakers' bureau of Foundation for Physician Advancement; is a paid employee for Stryker and Zimmer Biomet; receives Research support from a company or supplier as a Principal Investigator from Smith & Nephew; is a member of the Editorial Board of Journal of Arthroplasty; and is a board member or attends committee appointments at AAOS and AAHKS. Ran Schwarzkopf receives personal royalties from Smith & Nephew; is a paid consultant for Smith & Nephew (personal and institution), Intellijoint Surgical (personal and institution), and Zimmer Biomet (personal and institution); owns stock or stock options in PSI (personal) and Gauss Surgical (personal); receives research support as a principal investigator from Smith and Nephew (institution), Orthopaedic Research and Education Foundation (institution), AAHKS FARE Grant (institution); has Medical/Orthopaedic publications editorial/governing board for Arthroplasty Today and Journal of Arthroplasty (JoA); and is a board member or attends committee appointments at AAHKS Hip and Knee Society.

The other authors declare no potential conflicts of interest.

For full disclosure statements refer to https://doi.org/10.1016/j.artd.2025.101902.

## CRediT authorship contribution statement

**Kareem Omran:** Writing – review & editing, Writing – original draft, Visualization, Software, Methodology, Investigation, Formal analysis. **Colleen Wixted:** Writing – review & editing, Writing – original draft, Visualization, Validation, Investigation. **Daniel Waren:** Writing – review & editing, Visualization, Supervision, Project administration, Formal analysis. **Joshua C. Rozell:** Writing – review & editing, Visualization, Validation, Supervision, Project administration, Formal analysis. **Ran Schwarzkopf:** Writing – review & editing, Visualization, Validation, Supervision, Project administration, Formal analysis.

## Appendix A Supplementary data

Supplementary Table 1

Conflict of Interest Statement for Omran

Conflict of Interest Statement for Rozell

Conflict of Interest Statement for Schwarzkopf

Conflict of Interest Statement for Waren

Conflict of Interest Statement for Wixted

## References

[1] Pennington N., Redmond A., Stewart T., Stone M. (2014). The impact of surgeon handedness in total hip replacement. Ann R Coll Surg Engl.

[2] Healy W.L., Iorio R., Clair A.J., Pellegrini V.D., Della Valle C.J., Berend K.R. (2016). Complications of total hip arthroplasty: standardized list, definitions, and stratification developed by the hip society. Clin Orthop Relat Res.

[3] Kobayashi N., Yukizawa Y. (2023). Causes of failure after total hip arthroplasty: a narrative review of literatures. J Joint Surg Res.

[4] Domb B.G., El Bitar Y.F., Sadik A.Y., Stake C.E., Botser I.B. (2014). Comparison of robotic-assisted and conventional acetabular cup placement in THA: a matched-pair controlled study. Clin Orthop Relat Res.

[5] Snijders T., van Gaalen S.M., de Gast A. (2017). Precision and accuracy of imageless navigation versus freehand implantation of total hip arthroplasty: a systematic review and meta-analysis. Int J Med Robot.

[6] Ruangsomboon P., Ruangsomboon O., Osman K., Pincus D., Mundi R., Tomescu S. (2024). Clinical, functional, and radiological outcomes of robotic assisted versus conventional total hip arthroplasty: a systematic review and meta-analysis of randomized controlled trials. J Robot Surg.

[7] Kort N., Stirling P., Pilot P., Müller J.H. (2021). Clinical and surgical outcomes of robot-assisted versus conventional total hip arthroplasty: a systematic overview of meta-analyses. EFORT Open Rev.

[8] Kumar V., Patel S., Baburaj V., Rajnish R.K., Aggarwal S. (2021). Does robotic-assisted surgery improve outcomes of total hip arthroplasty compared to manual technique? A systematic review and meta-analysis. Postgrad Med J.

[9] Lim P.L., Gonzalez M.R., Wang K.Y., Sauder N., Bedair H.S., Melnic C.M. (2025). Does robotic assistance increase the likelihood of achieving the minimal clinically important improvement following total hip arthroplasty? Findings from a propensity score matched analysis of 1,364 procedures. J Arthroplasty.

[10] Dekhne M.S., Fontana M.A., Pandey S., Driscoll D.A., Lyman S., McLawhorn A.S. (2024). Defining patient-relevant thresholds and change scores for the HOOS JR and KOOS JR anchored on the patient-acceptable symptom state question. Clin Orthop Relat Res.

[11] Crosby R.D., Kolotkin R.L., Williams G.R. (2003). Defining clinically meaningful change in health-related quality of life. J Clin Epidemiol.

[12] Omran K., Schwarzkopf R. (2025). How should we define meaningful improvement? A commentary on minimal clinically important difference assessment for hip disability and osteoarthritis outcome score for joint replacement and knee injury and osteoarthritis outcome score for joint replacement in total joint arthroplasty. J Arthroplasty.

[13] von Elm E., Altman D.G., Egger M., Pocock S.J., Gøtzsche P.C., Vandenbroucke J.P. (2007). The strengthening the reporting of observational studies in epidemiology (STROBE) statement: guidelines for reporting observational studies. Epidemiology.

[14] Karunaratne S., Duan M., Pappas E., Fritsch B., Boyle R., Gupta S. (2019). The effectiveness of robotic hip and knee arthroplasty on patient-reported outcomes: a systematic review and meta-analysis. Int Orthop.

[15] Han P.F., Chen C.L., Zhang Z.L., Han Y.C., Wei L., Li P.C. (2019). Robotics-assisted versus conventional manual approaches for total hip arthroplasty: a systematic review and meta-analysis of comparative studies. Int J Med Robot.

[16] Lawrence K.W., Rajahraman V., Meftah M., Rozell J.C., Schwarzkopf R., Arshi A. (2024). Patient-reported outcome differences for navigated and robot-assisted total hip arthroplasty frequently do not achieve clinically important differences: a systematic review. HIP Int.

[17] Chen X., Xiong J., Wang P., Zhu S., Qi W., Peng H. (2018). Robotic-assisted compared with conventional total hip arthroplasty: systematic review and meta-analysis. Postgrad Med J.

[18] Zhang X., Shen X., Zhang R., Chen M., Ma R., Zhang Z. (2024). Radiographic evaluation of robot-assisted versus manual total hip arthroplasty: a multicenter randomized controlled trial. J Orthop Traumatol.

[19] Avram G.M., Prill R., Gurau C.D., Georgeanu V., Deleanu B., Russu O. (2023). Acetabular cup placement and offset control in robotic total hip arthroplasty performed through the modified anterolateral approach. Int Orthop.

[20] Kayani B., Tahmassebi J., Ayuob A., Konan S., Oussedik S., Haddad F.S. (2021). A prospective randomized controlled trial comparing the systemic inflammatory response in conventional jig-based total knee arthroplasty versus robotic-arm assisted total knee arthroplasty. Bone Joint J.

[21] Bini S.A., Fithian D.C., Paxton L.W., Khatod M.X., Inacio M.C., Namba R.S. (2010). Does discharge disposition after primary total joint arthroplasty affect readmission rates?. J Arthroplasty.

[22] Mahomed N.N., Davis A.M., Hawker G., Badley E., Davey J.R., Syed K.A. (2008). Inpatient compared with home-based rehabilitation following primary unilateral total hip or knee replacement: a randomized controlled trial. J Bone Joint Surg Am.

[23] Fu M.C., Samuel A.M., Sculco P.K., MacLean C.H., Padgett D.E., McLawhorn A.S. (2017). Discharge to inpatient facilities after total hip arthroplasty is associated with increased postdischarge morbidity. J Arthroplasty.

[24] Lyman S., Lee Y.Y., Franklin P.D., Li W., Mayman D.J., Padgett D.E. (2016). Validation of the HOOS, JR: a short-form hip replacement survey. Clin Orthop Relat Res.

[25] Rudisill S.S., Varady N.H., Birir A., Goodman S.M., Parks M.L., Amen T.B. (2023). Racial and ethnic disparities in total joint arthroplasty care: a contemporary systematic review and meta-analysis. J Arthroplasty.

[26] Kuitunen I., Ponkilainen V.T., Uimonen M.M., Eskelinen A., Reito A. (2021). Testing the proportional hazards assumption in cox regression and dealing with possible non-proportionality in total joint arthroplasty research: methodological perspectives and review. BMC Musculoskelet Disord.

[27] Gong Q., Fang L. (2013). Comparison of different parametric proportional hazards models for interval-censored data: a simulation study. Contemp Clin Trials.

[28] Omran K., Waren D., Schwarzkopf R. (2024). Postoperative pain trajectories in total hip arthroplasty. Bone Joint Open.

[29] Lewis G.N., Rice D.A., Rashid U., McNair P.J., Kluger M.T., Somogyi A.A. (2023). Trajectories of pain and function outcomes up to 5 to 8 years following total knee arthroplasty. J Arthroplasty.

[30] Sato E.H., Stevenson K.L., Blackburn B.E., Peters C.L., Archibeck M.J., Pelt C.E. (2023). Recovery curves for patient reported outcomes and physical function after total hip arthroplasty. J Arthroplasty.

[31] Jain D., Bendich I., Nguyen L.-C.L., Nguyen L.L., Lewis C.G., Huddleston J.I. (2017). Do patient expectations influence patient-reported outcomes and satisfaction in total hip arthroplasty? A prospective, multicenter study. J Arthroplasty.

[32] Salimy M.S., Paschalidis A., Dunahoe J.A., Chen A.F., Alpaugh K., Bedair H.S. (2024). Time to achieve the minimal clinically important difference in primary total hip arthroplasty: Comparison of anterior and posterior surgical approaches. J Arthroplasty.

[33] Nelms N.J., Birch C.E., Halsey D.H., Blankstein M., McGinnis R.S., Beynnon B.D. (2020). Assessment of early gait recovery after anterior approach compared to posterior approach total hip arthroplasty: a smartphone accelerometer-based study. J Arthroplasty.

[34] Zhao H.Y., Kang P.D., Xia Y.Y., Shi X.J., Nie Y., Pei F.X. (2017). Comparison of early functional recovery after total hip arthroplasty using a direct anterior or posterolateral approach: a randomized controlled trial. J Arthroplasty.

[35] Zawadsky M.W., Paulus M.C., Murray P.J., Johansen M.A. (2014). Early outcome comparison between the direct anterior approach and the mini-incision posterior approach for primary total hip arthroplasty: 150 consecutive cases. J Arthroplasty.

[36] Rodriguez J.A., Deshmukh A.J., Rathod P.A., Greiz M.L., Deshmane P.P., Hepinstall M.S. (2014). Does the direct anterior approach in THA offer faster rehabilitation and comparable safety to the posterior approach?. Clin Orthop Relat Res.

[37] Carender C.N., Hegde V., Levine B.R., Huddleston J.I., Cohen-Rosenblum A. (2025). Highlights of the 2024 American joint replacement registry annual report. Arthroplast Today.

