# Supplementary material for: Time to Achieve a Minimal Clinically Important Difference After Total Hip Arthroplasty: A Retrospective Cohort Comparison of Robotic-Assisted, Navigation-Assisted, and Conventional Techniques
Source: Arthroplast Today. 2025 Nov 8;36:101902. doi: 10.1016/j.artd.2025.101902 (PMC12648503; doi:10.1016/j.artd.2025.101902)
Supplement: Supplementary Table 1 [file mmc1.docx]

**Supplementary Table 1:** Akaike Information Criterion (AIC) values for different distributions considered for modelling the accelerated failure time models, stratified by method of Minimal Clinically Important Difference (MCID) calculation

| **Distribution** | **Distribution-Based MCID Threshold**  **AIC** | **Anchor-Based MCID Threshold**  **AIC** |
| --- | --- | --- |
| **Weibull** | 2225.42 | 2803.71 |
| **Exponential** | 2244.02 | 2891.36 |
| **Log-Normal** | 1951.28 | 2647.42 |
| **Log-Logistic** | 1934.01 | 2664.72 |

**Supplementary Table 2:** Time Ratios (TR) for Achieving a Minimal Clinically Important Difference (MCID) in HOOS-JR Scores by Patient, Procedure, and Surgical Approach, stratified by MCID Threshold.

| **Variable** | **Distribution-based Threshold** | | | **Anchor-based Threshold** | | |
| --- | --- | --- | --- | --- | --- | --- |
|  | TR | 95% CI | p-value | TR | 95% CI | p-value |
| **Pre-operative HOOS-JR Score** | 1.04 | 1.04–1.05 | <0.001 | 1.07 | 1.07–1.08 | <0.001 |
| **Procedure Type** |  |  |  |  |  |  |
| Conventional THA (reference) | – | – | – | – | – | – |
| Navigation-assisted THA | 0.88 | 0.74–1.06 | 0.151 | 1.08 | 0.87–1.34 | 0.477 |
| Robotic-assisted THA | 0.67 | 0.52–0.86 | 0.002 | 0.85 | 0.63–1.13 | 0.288 |
| **Age at Surgery** | 1.01 | 1.00–1.02 | 0.009 | 1.01 | 1.00–1.02 | 0.006 |
| **BMI** | 1.01 | 0.99–1.02 | 0.175 | 1.00 | 0.98–1.02 | 0.803 |
| **Sex** |  |  |  |  |  |  |
| Women (reference) | – | – | – | – | – | – |
| Men | 0.94 | 0.81–1.12 | 0.475 | 0.84 | 0.70–1.01 | 0.073 |
| **Marital Status** |  |  |  |  |  |  |
| Currently Partnered (reference) | – | – | – | – | – | – |
| Currently Single | 1.08 | 0.92–1.28 | 0.360 | 1.05 | 0.87–1.28 | 0.616 |
| Widowed | 0.94 | 0.70–1.24 | 0.675 | 0.79 | 0.56–1.14 | 0.183 |
| **Smoking Status** |  |  |  |  |  |  |
| Never Smoked (reference) | – | – | – | – | – | – |
| Current Smoker | 1.22 | 0.86–1.68 | 0.264 | 1.18 | 0.77–1.81 | 0.460 |
| Former Smoker | 1.03 | 0.87–1.23 | 0.686 | 1.09 | 0.90–1.32 | 0.356 |
| **ASA Physical Status** |  |  |  |  |  |  |
| 1–2 (reference) | – | – | – | – | – | – |
| 3–4 | 0.90 | 0.76–1.09 | 0.226 | 1.03 | 0.83–1.27 | 0.778 |
| **Race** |  |  |  |  |  |  |
| White (reference) | – | – | – | – | – | – |
| Black or African American | 1.25 | 0.97–1.56 | 0.082 | 1.31 | 0.97–1.77 | 0.071 |
| Asian | 1.65 | 1.02–2.67 | 0.042 | 1.20 | 0.65–2.22 | 0.573 |
| Other | 1.08 | 0.83–1.32 | 0.569 | 1.17 | 0.85–1.60 | 0.363 |
| Undeclared | 0.86 | 0.34–2.18 | 0.758 | 0.89 | 0.29–2.50 | 0.833 |
| **Anesthesia Type** |  |  |  |  |  |  |
| Regional (reference) | – | – | – | – | – | – |
| General | 1.25 | 0.95–1.65 | 0.117 | 1.26 | 0.90–1.77 | 0.189 |
| **Discharge Disposition** |  |  |  |  |  |  |
| Home (reference) | – | – | – | – | – | – |
| Facility-based Care | 1.77 | 1.17–2.67 | 0.008 | 1.19 | 0.70–1.93 | 0.509 |
| **Surgical Approach** |  |  |  |  |  |  |
| Anterior (reference) | – | – | – | – | – | – |
| Direct Lateral | 1.06 | 0.76–1.35 | 0.718 | 1.10 | 0.74–1.63 | 0.620 |
| Posterior | 0.87 | 0.68–1.12 | 0.234 | 1.06 | 0.81–1.39 | 0.690 |
| Mixed | 0.81 | 0.65–1.03 | 0.088 | 0.70 | 0.52–0.95 | 0.021 |

*Abbreviation key: MCID = Minimal Clinically Important Difference; HOOS-JR = Hip Injury and Osteoarthritis Outcome Score for Joint Replacement; THA = Total Hip Arthroplasty; TR = Time Ratio; CI = Confidence Interval; BMI = Body Mass Index; ASA = American Society of Anesthesiologists*
