# Supplementary material for: Time to Achieve a Minimal Clinically Important Difference After Total Hip Arthroplasty: A Retrospective Cohort Comparison of Robotic-Assisted, Navigation-Assisted, and Conventional Techniques
Source: Arthroplast Today. 2025 Nov 8;36:101902. doi: 10.1016/j.artd.2025.101902 (PMC12648503; doi:10.1016/j.artd.2025.101902)
Supplement: Conflict of Interest Statement for Rozell [file mmc3.docx]

# INDIVIDUAL CONFLICT OF INTEREST STATEMENT

***American Association of Hip and Knee Surgeons***

(Adopted from the American Academy of Orthopaedic Surgeons disclosure statement)

The following form **must be filled out completely and submitted by each author (example, 6 authors, 6 forms).**

**All items require a response. If there is no relevant disclosure for a given item, enter "*None*.”**

Manuscript Title: **Time to Achieve a Minimal Clinically Important Difference After Total Hip Arthroplasty: A Retrospective Cohort Comparison of Robotic-Assisted, Navigation-Assisted, and Conventional Techniques**

1. Royalties from a company or supplier (The following conflicts were disclosed)

None

2. Speakers bureau/paid presentations for a company or supplier (The following conflicts were disclosed)

Foundation for Physician Advancement

3A. Paid employee for a company or supplier (The following conflicts were disclosed)

None

3B. Paid consultant for a company or supplier (The following conflicts were disclosed)

Stryker, Zimmer Biomet

3C. Unpaid consultants for a company or supplier (The following conflicts were disclosed)

None

4. Stock or stock options in a company or supplier (The following conflicts were disclosed)

None

5. Research support from a company or supplier as a Principal Investigator (The following conflicts were disclosed)

Smith & Nephew

6. Other financial or material support from a company or supplier (The following conflicts were disclosed)

None

7. Royalties, financial or material support from publishers (The following conflicts were disclosed)

None

8. Medical/Orthopaedic publications editorial/governing board (The following conflicts were disclosed)

Journal of Arthroplasty – Editorial Board

9. Board member/committee appointments for a society (The following conflicts were disclosed)

AAOS, AAHKS

**Each author must sign AND print or type his/her name, date and submit a separate form**

In addition, one BLINDED Conflict of Interest form (no author names used) should be submitted per manuscript with all author disclosures.

Joshua Rozell, MD 2/4/25

Author Name (Print or Type) Author Signature Date
